# Protective and therapeutic role of mango pulp and eprosartan drug and their anti-synergistic effects against thioacetamide-induced hepatotoxicity in male rats

**DOI:** 10.1007/s11356-022-19383-9

**Published:** 2022-03-04

**Authors:** Nadia Zaki Shaban, Mohammad Mohammad Zaki, Fayed Koutb, Ahmed Alaa Abdul-Aziz, Ashraf Abdul-Hamid Elshehawy, Hany Mehany

**Affiliations:** 1 Biochemistry Department, Faculty of Science, Alexandria University, Alexandria, Egypt; 2 Chemistry Department, Faculty of Science, Kafrelsheikh University, Kafr El-Sheikh, Egypt; 3 Nucleic Acid Research Department, Genetic Engineering and Biotechnology Research Institute, City of Scientific Research and Technological Applications, Alexandria, Egypt; 4 Endocrinology Unit, Department of Internal Medicine, Faculty of Medicine, Alexandria University, Alexandria, Egypt

**Keywords:** Mango pulp, Phenolic and flavonoid compounds, Eprosartan, Hypertension, Thioacetamide, Rat hepatotoxicity, Oxidative stress, Hepatic inflammation, Liver fibrosis

## Abstract

The present study was done to evaluate the protective and therapeutic role of mango pulp (**M**), eprosartan drug (**E**), and their co-administration (**EM**) against hepatotoxicity induced by thioacetamide (**T**). Seven groups of rats were prepared as follows: the control **(C)** group (normal rats), **T** group (the rats were injected with T), **T-M** group (the rats were injected with T, and then treated with M), **T-E** group (the rats were injected with T, and then treated with E), **T-EM** group (the rats were injected with T, and then treated with E and M), **M-TM-M** group (the rats were administered with M before, during, and after T injection), and **M** group (the healthy rats were administered with M only). Firstly, the characterizations of **M** were determined. Also, the markers of hepatic oxidative stress [malondialdehyde (MDA) and glutathione (GSH) levels and the activities of superoxide dismutase (SOD), glutathione peroxidase (GPx), and glutathione reductase (GSR)], inflammation and fibrosis [(tumor necrosis factor-α (TNF-α) and platelet-derived growth factor-BB (PDGF-BB) levels and gene expression of transforming growth factor-beta1(TGF-β_1_)], and liver functions and microscopic examination were evaluated. The present results revealed that M contains 419 ± 1.04 μg total phenolics as gallic acid equivalent and 6.8 ± 0.05 μg total flavonoids as quercetin equivalent. The analysis of phenolics and flavonoids showed the presence of chlorogenic, caffeic, 2,5-dihydroxy benzoic, 3,5-dicaffeoylquinic, 4,5-dicaffeoylquinic, tannic, cinnamic acidS, and catechin, phloridzin, and quercetin with different concentrations. Also, M contains various minerals with different concentrations involving potassium, calcium, magnesium, sodium, iron, copper, zinc, and manganese. The current results showed that the total antioxidant capacity of 1 g of M was 117.2 ± 1.16 as μg ascorbic acid equivalent. Our biochemical studies showed that all treatments significantly reduced T-induced hepatotoxicity and liver injuries, as the oxidative stress and inflammatory and fibrotic markers were diminished where MDA level and the activities of GST, GSSG, and GR were decreased when compared with T group. In contrast, GSH level and the activities of SOD and GPx and GSH/GSSG ratio were increased. In addition, TNF-α and PDGF-BB levels were reduced, and the gene expression of TGF-β1 was down-regulated. Consequently, the liver functions were significantly improved. **In conclusion**, each **E**, **M**, and **EM** has a therapeutic effect against T-induced hepatotoxicity via the reduction of the OS, inflammation, and fibrosis. Unfortunately, treatment with **M** and **E** simultaneously revealed the less effectiveness than the treatment with **M** or **E** demonstrates the presence of anti-synergistic effect between them. Additionally, **M-TM-M** treatment showed a better effect than **T-M** treatment against T-induced hepatotoxicity revealing the prophylactic role of **M**. The administration of healthy rats with **M** for 12 weeks has no side effect.

**Graphical abstract:**

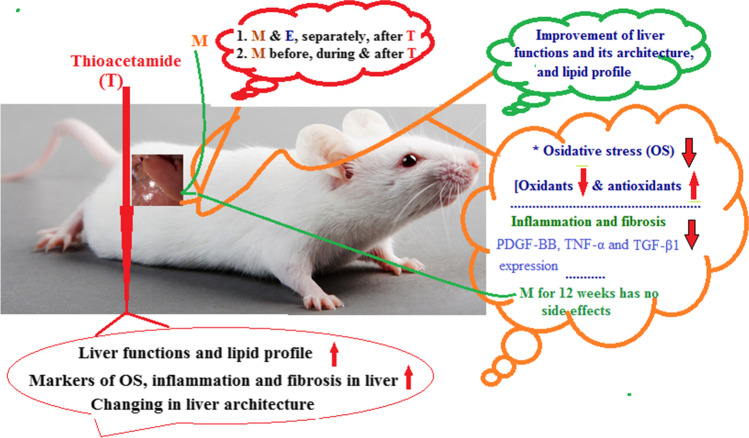

## Introduction

Liver has many metabolic, storing, and secretory functions. It secretes bile; forms blood clotting factors; metabolizes carbohydrates, proteins, and fats; stores vitamins, glycogen, and other substances; cleans blood from wastes and toxic matter; and removes old red blood cells (Koolman and Röhm 2005; Rodes et al. 2008). So, the liver is an important target of the toxicity of drugs and xenobiotics through generation of reactive oxygen species (ROS) and reactive nitrogen species (RNS) leading to the oxidative stress (OS) (Jaeschke et al. 2002). ROS and RNS in the hepatocytes are produced by some metabolic reactions such as in the process of mitochondrial oxidative phosphorylation or they may be produced from catabolism of xenobiotic compounds such as CCl_4_ and T (Shaban et al. 2021b, 2022). Cellular antioxidant defense system consists of enzymatic and non-enzymatic factors that maintain cellular redox homoeostasis. Enzymatic factors include superoxide dismutase (SOD), glutathione reductase (GSR), glutathione-S-transferase (GST), and glutathione peroxidase (GPx), while non-enzymatic involving reduced glutathione (GSH), carotenoids, polyphenols, and vitamins (Shaban et al. 2013, 2014, 2016a, b). The OS can occur, when ROS production overcomes the cellular antioxidant defense system whether via an increase in its production or a decrease in the cellular antioxidant ability (Friedman 1999; Saed et al. 2017). The OS performs in direct or indirect ROS-mediated damage of proteins, nucleic acids, and lipids, and has been implicated in diabetes, atherosclerosis, neurodegeneration, and carcinogenesis (Ray et al. 2012).

Thioacetamide (T) is known to produce marked hepatotoxicity in the exposed animals (Palacios et al. 2008). It is metabolized in the liver into active products, thioacetamide-S-oxide and thioacetamide-S,S-dioxide, which cause OS (Akhtar and Sheikh 2013). OS causes liver inflammation through the immune cells activation and production of pro-inflammatory and fibrotic cytokines such as transforming growth factor-beta 1 (TGF-β_1_), platelet-derived growth factor-BB (PDGF-BB), and tumor necrosis factor-α (TNF-α) (Friedman 1999; Saed et al. 2017; Shaban et al. 2017).

The expressing chemotherapy has come to point non-specific usage of intracellular poisons to suppress mitosis (cell division) or encourage DNA damage, which is why inhibition of DNA reform can increase chemotherapy. Many of the side impacts of chemotherapy can be traced to deterioration of normal cells which divide quickly consequently sensitive to anti-mitotic drugs (Rajman et al. 2018). In contrast, the medicinal plants have been used as a natural source of biologically active compounds with beneficial effects, no or less side effects and environmentally benign, which can be used to improve human health and their uses are increasingly recognized to prevent and treat many diseases (Hesari et al. 2021). Fruits are rich in phytochemicals that have been investigated for their potential health benefits. As a rich source of different biologically active compounds, mango (*Mangifera indica*) is an excellent example (Ma et al. 2011). Mango contains polyphenolics, flavonoids, and A and C vitamins. In addition, it contains some minerals which play a vital role in the body (Ma et al. 2011; Lauricella et al. 2017). Angiotensin II (Ang II) acts systemically to regulate blood pressure, electrolytes, and water homeostasis, through the two main receptors AT_1_ and AT_2_ (Forrester et al. 2018). Locally, it increases profibrogenic effects, inflammatory cells recruitment, cellular proliferation, and accumulation of extracellular matrix components through increasing secretion and activation of TGF-β_1_ via AT_1_ activation. Eventually, this leads to tissue injury (Forrester et al. 2018).

**Fig. 1:**
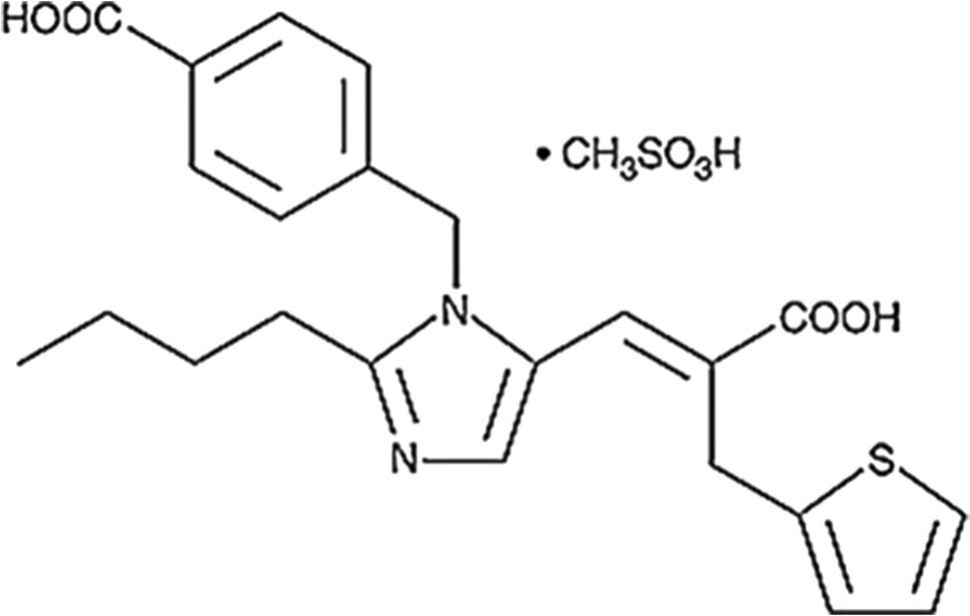
Chemical structure of eprosatan mesylate

Eprosartan mesylate drug (E, Fig. 1) is a dicarboxylic acid, a member of the class of imidazoles and thiophenes (Tenero et al. 1998). It is a competitive and reversible angiotensin II receptor antagonist with anti-hypertensive property (Frishman et al. 2005). It blocks the binding of angiotensin II to AT_1_ receptor in vascular smooth muscle, that way blocking the principal action of angiotensin II on the renin-angiotensin system leading to vascular dilatation (WONG et al. 2000). E is not metabolized by the cytochrome P450 system. It is mainly eliminated as unchanged drug. Less than 2% of an oral dose is excreted in the urine as an acyl glucuronide of **E** (Frishman et al. 2005).

In the current study, we evaluated the therapeutic effect of mango pulp (**M**), E, and their co-administration against T-induced hepatotoxicity in rats. The study focused on the determination of the markers of OS, inflammation, and fibrosis in the liver and liver functions. Also, liver microscopic examination was done. The phytochemical compositions of **M** especially phenolic and flavonoid contents and minerals were evaluated.

## Material and methods

### Chemicals

PDGF-BB and TNF-α ELISA kits were purchased from, R & D Systems, USA, and eBioscience Bender MedySystems GmbH, respectively. Isolate II RNA mini kit and quantitative real-time polymerase chain reaction (qRT-PCR) kit (Sensifast SYBR No-ROX one step kit) were purchased from Bioline, London. TGF-ß1 and glyceraldehyde 3-phosphate dehydrogenase (GAPDH) primers were purchased from Bio basic Inc., Canada. T and E were purchased from Oxford Lab Chem, India, and Abbott Healthcare SAS, France, respectively. Gallic acid, ascorbic acid, and quercetin were purchased from Sigma–Aldrich, USA. Kits for alanine transaminase (ALT), aspartate transaminase (AST), gamma-glutamyl transpeptidase (GGT), bilirubin, albumin, total protein (TP), urea, and creatinine were purchased from spectrum diagnostics, Egypt. Kits of SOD, GSH, GST, and malondialdehyde (MDA) were purchased from Biodiagnostic, Egypt. Kit for assays of GPx was obtained by Gentaur’s kits, Belgium. Kit of GSR was purchased from Cayman’s kits, USA.

### Fruit collection, extract preparation, and characterization

Fruit collection: Mango fruit (*Mangifera indica* L., family Anacardiceae), Langra cultivar, was obtained during the month of September 2014 from a local market at Alexandria, Egypt. The healthy fruits of uniform size and appearance were washed, and the pulp of each fruit was cut and stored at – 20 °C until used.

Extract preparation: The extract was prepared according to the procedure of Kim et al. (2010), where mango pulp (M) was lyophilized for 2 days and ground and the powder was extracted with 5 volumes (w/v) of 80% ethanol by sonication for 3 days at room temperature. The extract was filtered, and the filtrate was concentrated using rotatory evaporator (Heidolph Hei-VAP Platinum) at 40 °C and lyophilized and stored at – 80 °C until used.

### Total phenolic and flavonoid contents in mango pulp

The total phenolic content was determined, using gallic acid as standard (Taga et al. 1984). In brief, 100 μl Folin-Ciocalteau reagent and 2 ml of 2% Na_2_CO_3_ were added to 100 mg of the extract and incubated for 2 h at room temperature. The absorbance of the resulting blue color was measured at 750 nm. The total phenolic content was expressed in milligrams of gallic acid equivalents per gram M.

Total flavonoid content was determined, using quercetin as standard (Zou et al. 2004). Briefly, 0.5 g of the extract was solubilized in 2 ml of distilled water and 0.15 ml of 5% sodium nitrite solution and incubated at room temperature for 6 min. Then, 0.15 ml of 10% aluminum chloride solution was added and left to stand for 6 min, followed by adding 2 ml of 4% NaOH solution to the mixture. The mixture was made up to 5 ml with ethanol. The absorbance was measured at 510 nm after incubation for 15 min. The total flavonoid content was expressed in milligrams of quercetin equivalents per gram M

### Analysis of phenolic and flavonoid using high-performance liquid chromatography (HPLC)

Phenolic and flavonoid were analyzed by HPLC using standard phenolic and flavonoid compounds. In brief, 200 mg of the extract was dissolved in 1 ml of 80% ethanol to prepare extract solution, where 20 μl of it was analyzed using Eclipse XDB-C18 (5 μm, 4.6 × 150 mm) column. The mobile phase was consisted of 1% (v/v) formic acid in aqueous solution: acetonitrile: 2-propanol (70:22:8), pH 2.5; at 0.75 ml/min flow rate, UV detection at 320 nm; Agilent technologies 1200S.

### Mango pulp total antioxidant capacity

It was determined by phosphomolybdenum method of Prieto et al. (1999) using ascorbic acid as standard and expressed in terms of ascorbic acid equivalent, where 300 μl of the ethanolic extract solution, which prepared as mentioned above, was mixed with 2.7 ml of the reagent solution (0.6 M sulfuric acid, 28 mM sodiumphosphate, and 4 mM ammonium molybdate). For the blank, 0.3 ml methanol was mixed with 2.7 ml of the reagent. The absorbance of the sample was measured at 695 nm.

### Mango pulp minerals contents

The minerals in M were determined according to the method of Ajai et al. (2014) using flame atomic absorption spectroscopy (Avanta Ʃ GBC GF 3000), where a standard solution containing a known concentration of the metal of interest is aspirated into the burner (air/acetylene). Briefly, M was homogenized well in two volumes (w/v) of 70% nitric acid in a beaker and the homogenate was heated at 90 °C on a hot plate in a fume hood for 1 h. Then, it was allowed to cool, filtered, and then diluted to 50 ml with distilled water and stored in a polyethylene container until analysis.

### Animals

Seventy healthy male Sprague-Dawley rats weighing from 90 to 100 g with age of about 3 months were obtained from the Medical Technology and Research Centre, Alexandria University, Egypt. The rats were tested for normal health status and kept for 2 weeks for laboratory environment acclimatization. The rats were allowed free access to normal rat’s food and tap water and were kept under conventional conditions of temperature, humidity, and light/dark cycle.

### Experimental design

**Fig. 2:**
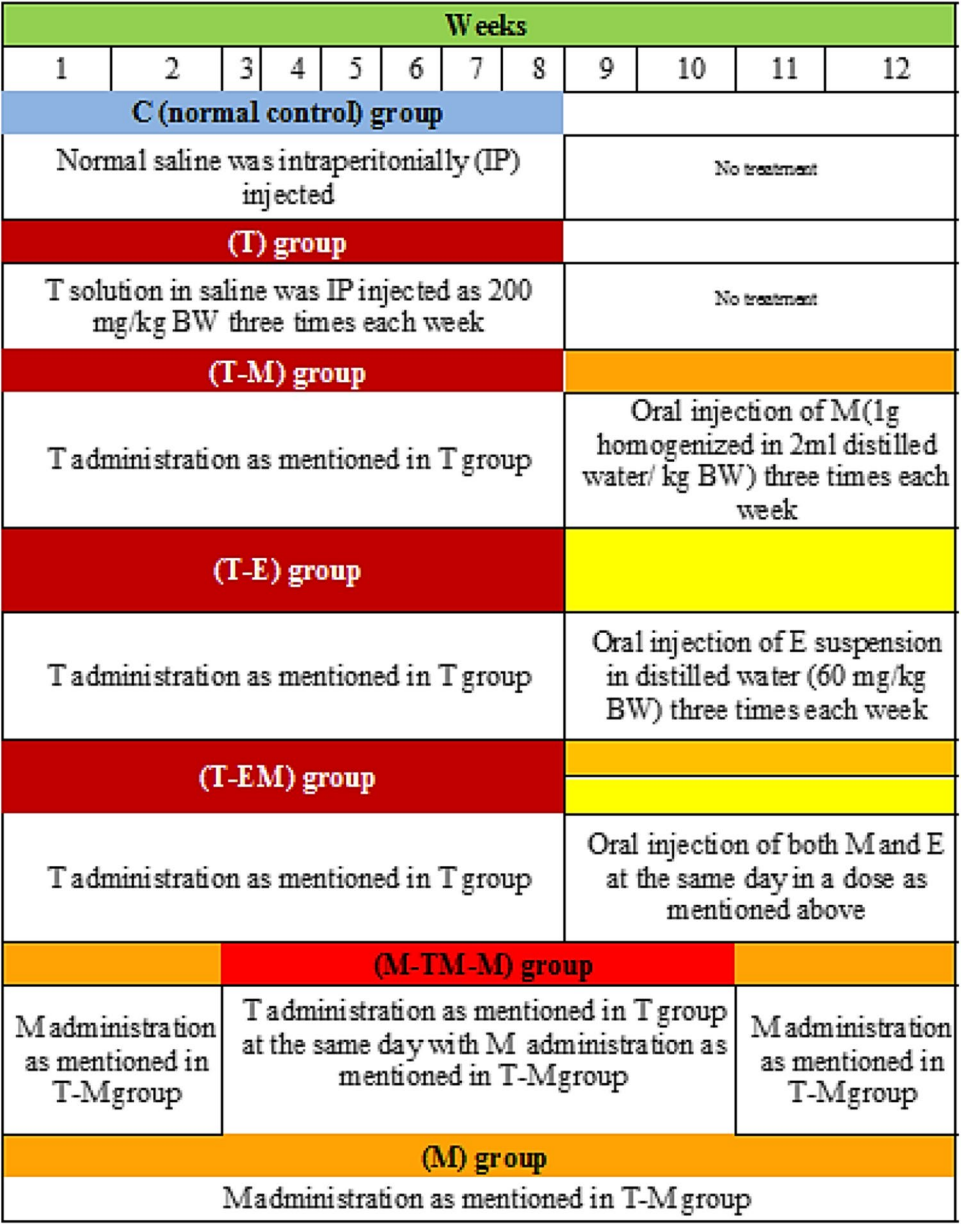
An illustration of the experimental design. All animals received the normal diet throughout the whole experimental period

The rats were divided into seven groups (Fig. 2), *n* = 10 for each, including, the control** (C)** group, normal rats where the rats were intraperitonially (IP) injected with saline (1 ml/kg body weight (BW) three times per week for eight successive weeks; **T** group, the rats were IP injected with **T** (200 mg dissolved in 1 ml saline/kg BW), three times per week for eight successive weeks (Morsy et al. 2015; Wallace et al. 2015); **T-M** group, the rats were injected with **T** as in **T** group; then at the beginning of the 9th week, the rats were orally administered with 1g of M/kg BW (**M** was homogenized with 2 ml distilled water) three times weekly for four successive weeks (Lucas et al. 2011); **T-E** group, the rats were injected with T; then at the beginning of the 9th week, they were administered with 60 mg of E/kg BW (E was suspended in 1ml distilled water) three times weekly for four weeks (Morsy et al. 2015); **T-EM** group, the rats were injected with T; then at the beginning of the 9th week, they were administered with both M and E, at the same day, respectively, three times weekly for four successive weeks; **M-TM-M** group, the rats were administered with M before (for 2 weeks), during, and after (for 2 weeks) T injection; and **M** group, M was administered three times per week for 12 weeks.

At the end of the experimental period (12 weeks), the rats were fasted overnight, weighted, anaesthetized by carbon dioxide, and sacrificed. Blood samples were collected from caudal vena cava, kept at room temperature for 15 min, and then centrifuged at 3000 rpm for 10 min to obtain serum, which stored at – 20 °C until used. The livers were removed quickly; small parts of each were fixed in 10% formalin for microscopic examination. The remaining parts were washed with normal saline, divided into parts, and kept immediately at – 80 °C until used.

### Biochemical assays

#### Markers of liver functions

In serum, AST, ALT, GGT, bilirubin, albumin, and total protein (STP), beside hepatic albumin and total protein (LTP), were determined using kit (Malloy and Evelyn 1937; Gornall et al. 1949; Reitman and Frankel 1957; Szasz 1974; Tietz NaML 1987)**.**

#### Hepatic oxidative stress markers

The liver homogenates were prepared by homogenizing the liver tissues in 10 volumes (w/v) of potassium phosphate buffer (0.1 M, pH 7.5) using a glass–Teflon homogenizer (Shaban et al. 2014). The homogenates were centrifuged at 4000 rpm for 20 min at 4 °C, and the supernatants were kept at − 80 °C until used for determination of the levels of MDA and GSH as well as the activities of SOD, GST, GPx, and GR. The MDA levels were determined using kit and expressed as nmol/mg tissue protein (Ohkawa et al. 1979). Total GSH and GSH were determined using kit and expressed as mg/mg tissue protein (Sedlak and Lindsay 1968). Then, the GSSG levels were calculated from the equation: (GSSG = Total GSH − reduced GSH). The redox status was demonstrated as GSH/GSSG ratio. The activities of GSR were determined using kit and expressed as nmol of NADPH consumed/mg tissue protein/min (Carlberg and Mannervik 1985). The SOD activities were determined using kit and expressed as U/mg tissue protein (Michiels et al. 1994). Total GST activities were measured using kit and expressed as nmol 1-chlor 2,4-dinitrobenzene (CDNB) conjugation/min/mg tissue protein (Habig et al. 1974). Total GPx activities were determined using kit and expressed as μmol NADPH consumed/min/mg tissue protein (Paglia and Valentine 1967).

### The inflammatory and fibrotic markers

#### TNF-α and PDGF-BB

Serum levels of TNF-α and PDGF-BB of the different studied groups were determined as pg/mg tissue protein, according to instructions of ELISA kits.

#### TGF-β1 expression assay

Hepatic RNA of each rat was isolated according to the instructions of the kit. The purity of extracted RNA samples was tested by absorbance of RNA samples at 260 and at 280 nm ratio (A260/A280). RNA concentration was measured using spectrophotometer (BioDrop μLite, Australia), while its quality was confirmed by gel electrophoresis on 2% agarose gel which was stained with ethidium bromide. In the isolated RNA samples, TGF-ß1 expression levels were determined through quantitative reverse transcriptase PCR technique using SYBR green PCR master mix one-step kit. Briefly, in a 20 μl reaction volume, 4 μl of the template (RNA sample) was added to 10 μl of 2× SYBR mix, 0.8 μl of each 10 μM forward and reverse primers, 0.4 μl RNase inhibitor, 0.2 μl reverse transcriptase, and 3.8 μl RNase-free water. The reactions included one cycle 10-min reverse transcription at 45 °C, one cycle 2 min of polymerase activation at 95 °C, and 40 cycles of 5s denaturation at 95 °C, 10 s annealing at 60 °C and 72 °C extension. The expression level all studied groups were calculated from the PCR cycle number where the increased fluorescence curve passes across a threshold cycle (CT). The relative expression of TGF-ß1 gene was obtained using comparative CT (ΔΔCT) method, and GAPDH gene was used as internal control. ΔCT and ΔΔCT were calculated by following equations: ΔCT = CT _(TGF-ß1)_ – CT _(GAPDH)_ and ΔΔCT = ΔCT _(Sample)_ – ΔCT _(Control)_. The expression fold changes were calculated from this formula: Expression fold change = 2 ^– ΔΔCT^ (Livak and Schmittgen 2001). The following primers were used (Table 1): *TGF–β1* (GI: 57341) forward primer (5′-TGGCGTTACCTTGGTAACC-3′), reverse primer (5′-GGTGTTGAGCCCTTTCCAG-3′), *GAPDH* (GI: 10190788) forward primer (5′-AGCCCAGAACATCATCCCTG-3′), reverse primer (5′-CACCACCTTCTTGATGTCATC -3′) (Wickert et al. 2002).

**Table 1:** The primers in qRT-PCR analysis

Gene	Primer name	Sequence (5′-3′)
*TGF–β1*(GI: 57341)	Forward	TGGCGTTACCTTGGTAACC
	Reverse	GGTGTTGAGCCCTTTCCAG
*GAPDH* (GI: 10190788)	Forward	AGCCCAGAACATCATCCCTG
	Reverse	CACCACCTTCTTGATGTCATC

#### Drug combination index (CI) analysis

The drug and food combination may offer greater (synergistic), lesser (anti-synergistic), or no new (additive) outcome compared to the single agent. This result can be investigated by calculating the CI value (the predictable value divided by the observed value for T-EM). The effect of the co-administration (EM) on predictable values of major parameters was determined as [(observed value for T-M)/(T value)] × [(observed value for T-E)/(T value)] × (T value) [34]. From the obtained CI values, the effect can be evaluated as synergism (CI < 1), anti-synergism (CI > 1), or additive (CI = 1) (Zhou et al. 2007; Habashy et al. 2018; Shaban et al. 2020, 2021a).

#### Hepatic microscopic examination

The liver samples were embedded in paraffin, sectioned at 5 μm thickness, and stained with hematoxylin and eosin (H & E) for microscopic examination (Suzuki and Suzuki 1998).

#### Statistical analysis

All data were given as means ± SD. Comparisons between the means of studied groups were performed by Tukey’s test (ANOVA) using the statistical software SPSS 16.0 V (SPSS, Inc., Chicago, IL, USA), and the differences were considered significant at (*P≤ 0.05*).

## Results

### Total phenolics and total flavonoids and HPLC analysis

The results showed that 1 g of M contains 419 ± 1.04 μg total phenolics as gallic acid equivalent and 6.8 ± 0.05 μg total flavonoids as quercetin equivalent. Also, the results in Table 2 show that M contains chlorogenic, caffeic, 2,5-dihydroxy benzoic, 3,5-dicaffeoylquinic, 4,5-dicaffeoylquinic, tannic acid, cinnamic acid, catechin, phloridzin, and quercetin with different concentrations.

**Table 2:** Mango pulp phenolics, flavonoids, and minerals contents

Phenolics/flavonoids	Concentration (μg/100 g)	Minerals	Concentration (mg/100 g)
Catechin	278.4	K	142.4
Quercetin	266.5	Ca	20.8
3,5-Dicaffeoyl quinic acid	100.8	Mg	3.2
Cinnamic acid	80.0	Na	2.4
Chlorogenic acid	73.7	Fe	0.8
2,5-Dihydroxy benzoic acid	53.6	Cu	0.2
Phloridzin	26.8	Zn	0.2
4,5-Dicaffeoyl quinic acid	24.8	Mn	0.1
Caffeic acid	13.6		
Tannic acid	7.2		

### Minerals

The results revealed that M contains different concentrations from potassium, calcium, magnesium, sodium, iron, copper, zinc, and manganese (Table 2).

### Total antioxidant capacity

The current results showed that the total antioxidant capacity of 1 g of M was 117.2 ± 1.16 as μg ascorbic acid equivalent.

### Different treatments improved liver dysfunctions induced by T

The results in Table 3 show that T injection caused significant (*P* < 0.001) elevations in the levels of ALT, AST, GGT, and bilirubin as compared to the C group, whereas LTP, STP, and albumin levels were reduced. In contrast, ALT, AST, GGT, and bilirubin of rats treated by **M**, **E**, and **ME** after **T** injection or **M-TM-M** became lower than the T group, while LTP, STP, and albumin levels were greater. In comparison with C group, M group results showed non-significant changes. Generally, ME treatment gave lower effect than the treatment with M and E separately, as well as M-TM-M treatment showed better effect than M treatment.

**Table 3:** Effect of different treatments on liver functions

Studied groups							
Parameters	C	T	T-M	T-E	T-EM	M-TM-M	M
In serum:							
ALT (U/L)	74.8 ± 3.2	128.5^a^ ± 1.9	102.2^a,b^ ± 2.7	77.8 ^b,c^ ± 1.0	99 ^a,b^ ± 2.0	81.0^a, b, c^ ± 3.7	76.1^b,c^ ± 0.5
AST (U/L)	107.9 ± 2.1	191.3^a^ ± 3.8	141^a,b^ ± 0.9	110.8 ^b,c^ ± 0.3	145.9 ^a,b,c,d^ ± 1.9	117.5^a,b,c^ ± 3.7	110.6^b,c^ ± 2.7
GGT (U/L)	3.0 ± 0.2	14.7^a^ ± 0.5	5.5^a,b^ ± 0.2	4.1 ^a,b,c^ ± 0.2	8.5 ^a,b,c,d^ ± 0.2	4^a,b,c^ ± 0.2	3.3^b,c^ ± 0.1
T.bil (mg/dl)	0.3 ± 0.0	0.9^a^ ± 0.0	0.5^a,b^ ± 0.0	0.33 ^a,b,c^ ± 0.02	0.48 ^a,b,d^ ± 0.01	0.4^a,b,c^ ± 0.02	0.25^b,c,d^ ± 0.03
Alb (g/dl)	3.9 ± 0.2	2.5^a^ ± 0.2	2.8^a^ ± 0.2	3.4^b,c^ ± 0.3	3.6 ^b,c^ ± 0.1	3.4^a,b,c^ ± 0.3	3.6^b,c^ ± 0.1
T.P(g/dl)	5.7± 0.3	3.5^a^ ± 0.2	4.1^a^ ± 0.5	5 ^a,b,c^ ± 0.1	3.9^a,d^ ± 0.4	4.9^a,b,c^ ± 0.3	5.5^b,c,d^ ± 0.1
In liver:							
Alb (g/g. liver)	6.2 ± 0.3	3.3^a^ ± 0.2	4.8^a, b^ ± 0.3	4.8^a, b, c^ ± 0.1	3.8^a,b,c,d^ ± 0.3	271.7^a, b, c^ ± 17.1	297.2^b,c,d^ ± 4.0
T.P (g/g. liver)	7.7 ± 0.1	4.1^a^ ± 0.1	6.6^a, b^± 0.5	6.3^a, b^ ± 0.3	5.4^a,b,c,d^ ± 0.2	341.7^a, b^ ± 14.9	385.2^b,c,d^ ± 14.9

*C* control rats; *T* rats were injected with thioacetamide; *T-M* rats were treated by mango pulp (M) after T injection; *T-E* rats were treated by eprosartan (E) after T injection; *T-EM* rats were treated by both E and M; *M-TM-M group* rats were treated by M before, during, and after T; and *M group* rats were treated by M only. *ALT* (alanine transaminase), *AST* (aspartate transaminase), *GGT* (gamma-glutamyl transpeptidase), *T.bil* (total bilirubin), *Alb* (albumin), and *T.P* (total protein). Since a significant (*p* < 0.05) vs C group; b significant (*p* < 0.05) vs T group and c significant (*p* < 0.05) vs T-M group, d significant (*p* < 0.05) vs T-E group, *n* = 10 per group, and the data are being presented as the mean ± SD

### Different treatments reduced OS induced by T

**Fig. 3:**
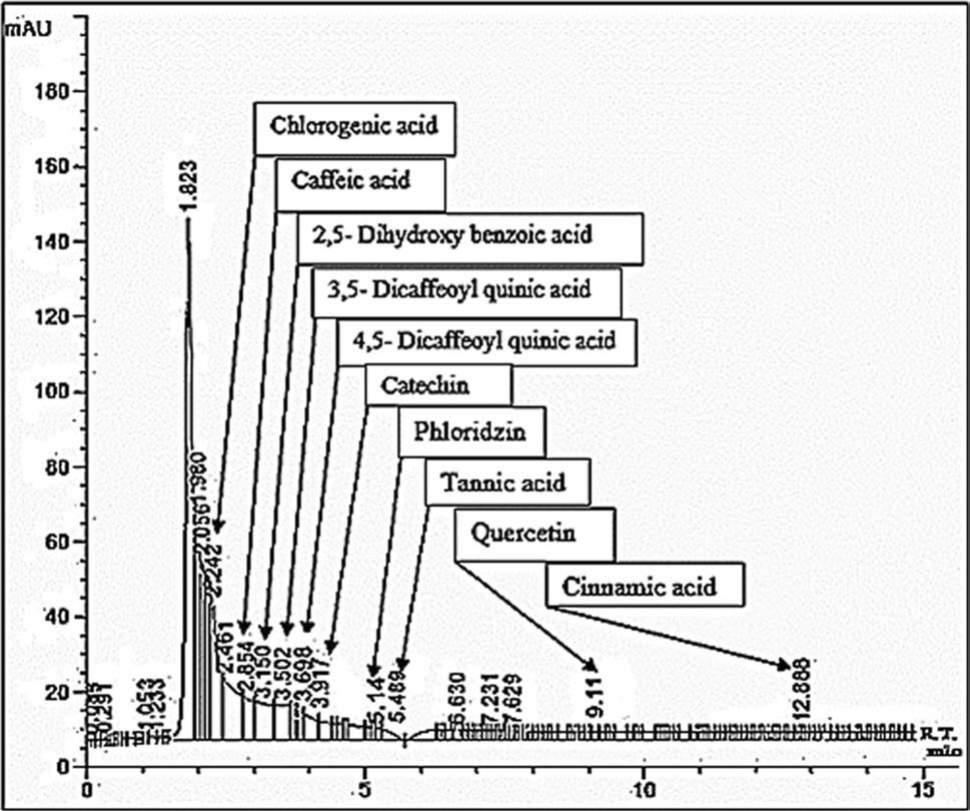
HPLC analysis of phenolics and flavonoids in M pulp

**Fig. 4:**
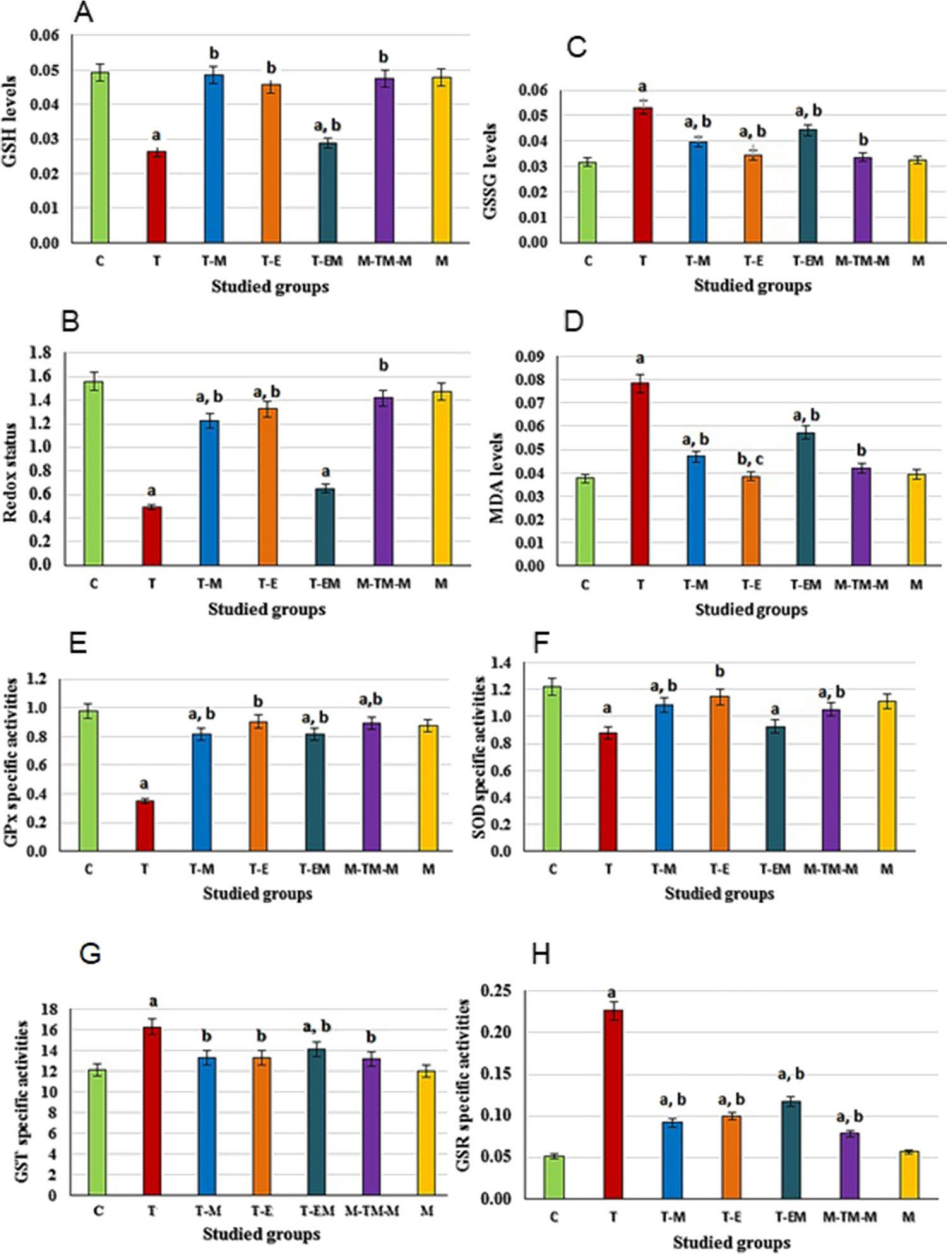
Effect of different studied treatments on the oxidative stress markers, where C: control rats; T: rats were injected with thioacetamide; T-M: rats were treated by mango pulp (M) after T injection; T-E: rats were treated by eprosartan (E) after T injection; T-EM: rats were treated by both E and M after T injection; M-TM-M group: rats were treated by M before, during, and after T; and M group: rats were treated by M only. Since a significant (*p* < 0.05) vs C group; b significant (*p* < 0.05) vs T group and c significant (*p* < 0.05) vs T-M group, d significant (*p* < 0.05) vs T-E group, *n* = 10 per group, and the data are being presented as the mean ± SD. GSH and GSSG levels are being expressed as mg/mg protein and MDA as nmol/mg protein; GPx-specific activities are being expressed as μmol NADPH/min/mg protein, SOD as U/mg protein, GST as μmol CDNB/min/mg protein, and GSR as nmol NADPH/min/mg protein

The results in Figs. 3 and 4 show that T injection caused significant (*P* < 0.001) elevations in the levels of MDA and GSSG and the activities of GST and GSR as compared with the C group, but the antioxidants (GSH, GPx and SOD) and redox status level were significantly (*P* < 0.001) declined. However, M, E, or ME treatments after T injection, as well as M-TM-M, significantly reduced the levels of MDA, GSSG, GST, and GSR when compared with the T group, although GSH levels, GPx, and SOD activities, and redox status levels were increased. In addition, results after only M administration (M group) showed non-significant changes in these parameters as compared with C group. Of note, ME treatment gives lower effect than the treatment with M and E separately (anti-synergistic effect) (Table 4).

**Table 4:** The combination index (CI)* values for the mango pulp (M) and eprosartan drug (E) co-administration on major tested parameters

Parameters	CI*	Effect
GSH	2.9 ± 0.1	Anti-synergistic
GSSG	1.7 ± 0.0	Anti-synergistic
GSH/GSSG	5.1 ± 0.3	Anti-synergistic
GPx	2.6 ± 0.2	Anti-synergistic
SOD	1.5 ± 0.1	Anti-synergistic
GST	1.3 ± 0.0	Anti-synergistic
GSR	2.9 ± 0.2	Anti-synergistic
MDA	2.5 ± 0.2	Anti-synergistic
PDGF-BB	1.9 ± 0.1	Anti-synergistic
TNF-α	2.4 ± 0.4	Anti-synergistic
TGF-β1	2.3 ± 0.2	Anti-synergistic

*GSH* (glutathione), *GSSG* (reduced glutathione), *GPx* (glutathione peroxidase), *SOD* (superoxide dismutase),* GST* (glutathione-S-transferase), *GSR* (glutathione reductase), *MDA* (malonedialdehyde), *TNF-α* (tumor necrosis factor-α), and PDGF-BB (platelet-derived growth factor-BB), and *TGF-β1* (transforming growth factor-beta1 and the data are being presented as the mean ± SD

^*^ CI values determine the type of impact between E and M. the effect can be evaluated as synergism (CI < 1), anti-synergism (CI > 1), or additive (CI = 1) (Zhou et al. 2007; Habashy et al. 2018; Shaban et al. 2020, 2021a, 2022)

### Different studied treatments reduced liver inflammation and fibrosis induced by T

**Fig. 5:**
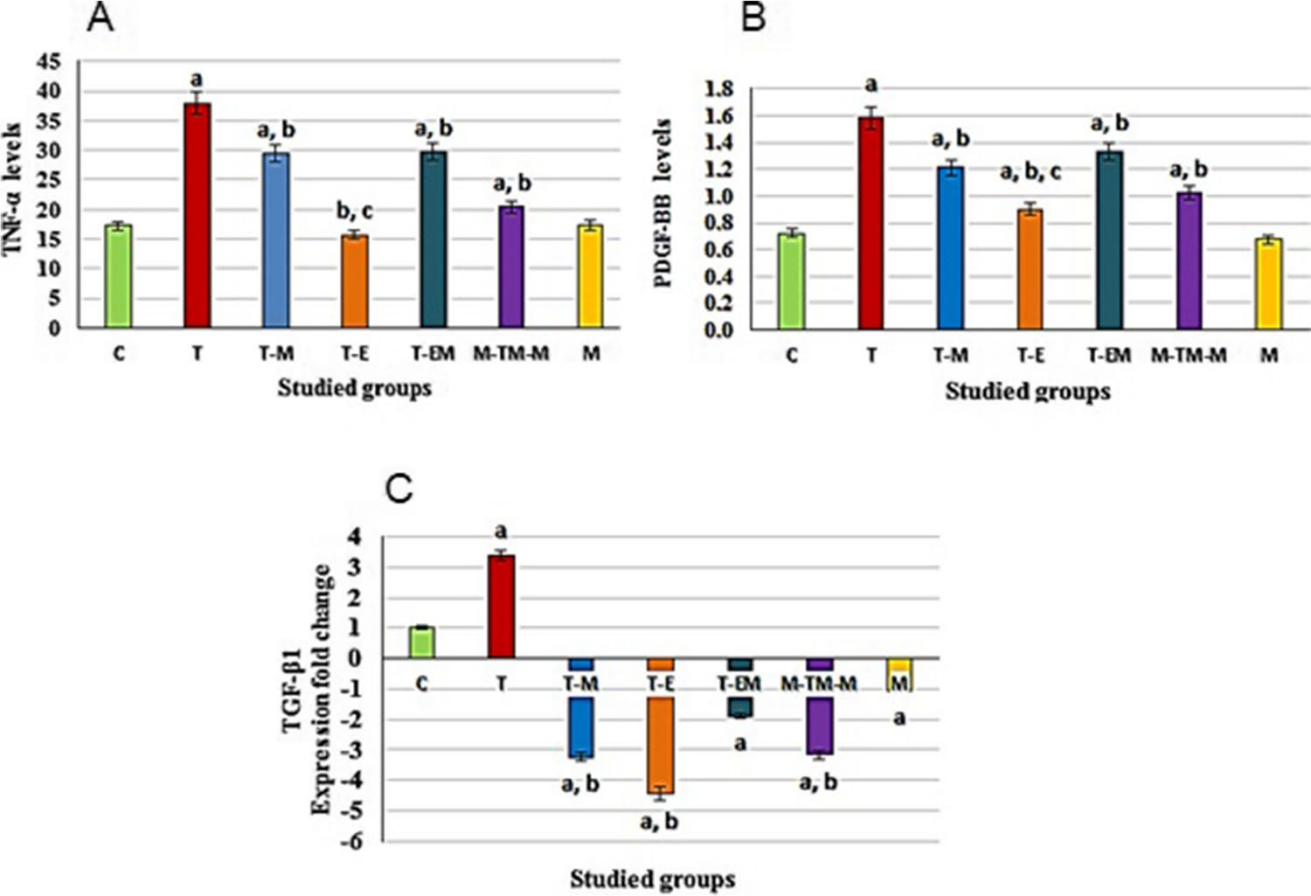
Effect of different studied treatments on hepatic inflammatory and fibrotic markers, where C: control rats; T: rats were injected with thioacetamide; T-M: rats were treated by mango pulp (M) after T injection; T-E: rats were treated by eprosartan (E) after T injection; T-EM: rats were treated by both E and M after T injection; M-TM-M group: rats were treated by M before, during, and after T; and M group: rats were treated by M only. TNF-α (tumor necrosis factor-α) and PDGF-BB (platelet-derived growth factor-BB) levels are being expressed as pg/mg protein, and TGF-β1 (transforming growth factor-beta1) expressed as fold change using *GAPDH* gene as housekeeping gene. Since a significant (*p* < 0.05) vs C group; b significant (*p* < 0.05) vs T group and c significant (*p* < 0.05) vs T-M group, d significant (*p* < 0.05) vs T-E group, *n* = 10 per group, and the data are being presented as the mean ± SD

The results in Fig. 5 reveal that T administration caused significant (*P* < 0.001) elevations in the levels of TNF-α and PDGF-BB beside significant (*P* < 0.05) up-regulation of the TGF-ß1 gene expression level as compared with the C group. However, treatment with M, E, ME, or M-TM-M after T injection significantly decreased these markers as compared to the T group. Moreover, the M group showed non-significant changes in results of these parameters as compared with the results of the C group. Noteworthy, ME treatment has a lower effect than the treatment with M and E separately as shown in Table 4.

### Different studied treatments improved liver histopathology induced by T

**Fig. 6:**
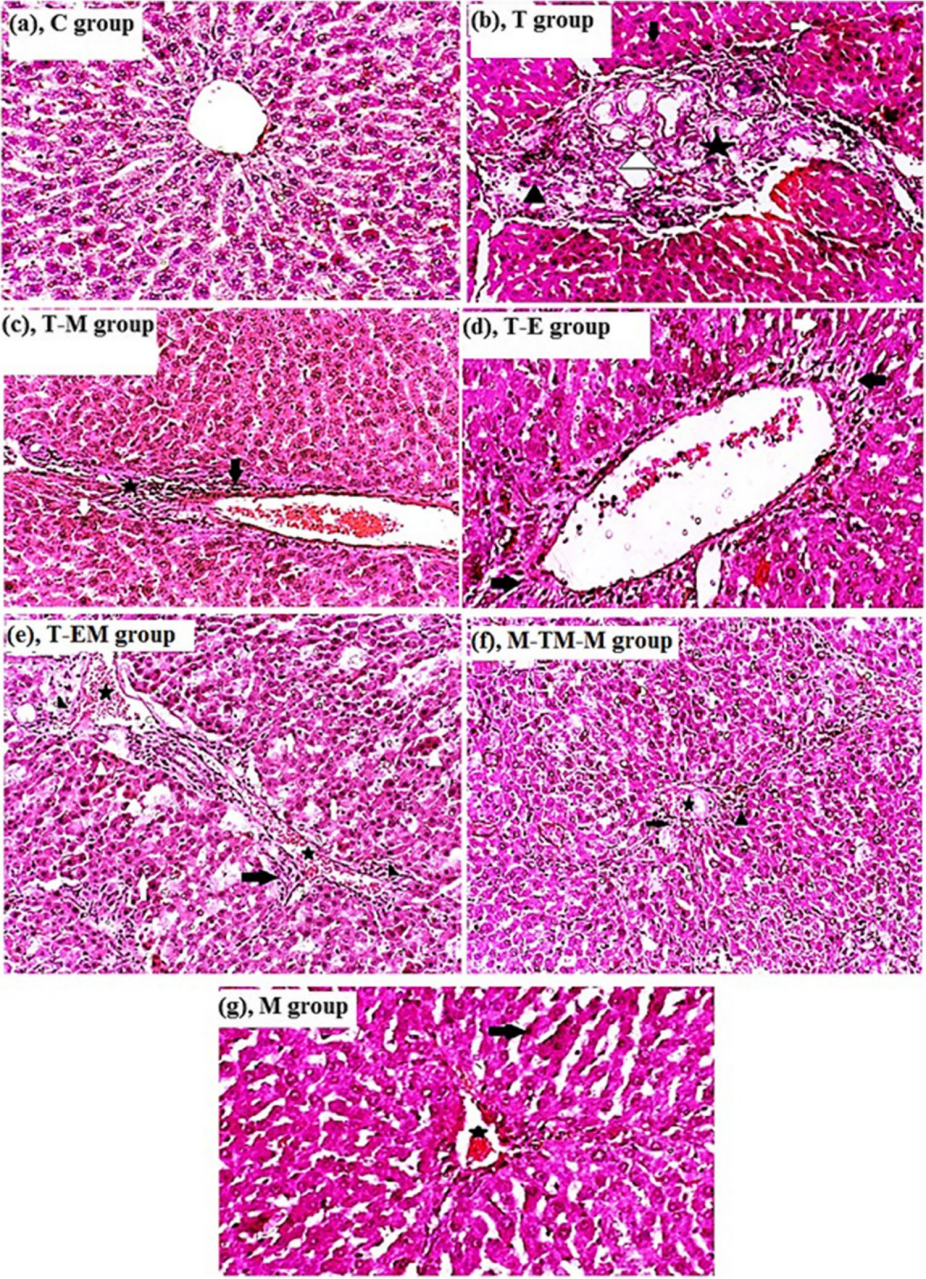
Effect of different studied treatments on the microscopic examination of liver sections (H & E staining; 20×), where (**a**) the normal control, **C group**, the liver sections showing normal central vein surrounded by normal hepatocytes which arranged in cords and separated by blood sinusoids); (**b**) **T group**, rats were injected with thioacetamide (T) showing excessive periportal and inter lobular fibrosis (black star), loss of general architecture of hepatic lobules, mononuclear cell infiltration (black arrowhead), binucleated hepatocytes (black arrow), bile duct hyperplasia (white arrowhead) and pyknotic nuclei undergoing apoptosis (white arrow); (**c**) **T-M group**, rats were treated by mango pulp (M) after T injection showing a small number pyknotic nuclei undergoing apoptosis (white arrow) and a little periportal degeneration of hepatocytes (black arrow) with mononuclear cells infiltration and myofibroblasts (black star); (**d**) **T-E group**, rats were treated by eprosartan (E) showing no fibrosis but there were small number of pyknotic nuclei undergoing apoptosis (white arrow) and pericentral hepatocyte degeneration (black arrow); (**e**) **T-EM group**, rats were simultaneously treated by both E and M after T injection showing periportal fibrosis (black arrow), pyknotic nuclei (white arrowhead), blood vessel congestion (black star), binucleated hepatocytes (white arrow) and mononuclear cell infiltration (black arrowhead); (**f**) **M-TM-M group**, rats were treated by M before, during, and after T injection showing pyknotic nuclei (white arrow), blood vessel congestion (black star), and few centrilobular degeneration of hepatocytes (black arrow) and mononuclear cells infiltration (black arrowhead); and (**g**) **M group**, rats were treated by M only showing only blood vessel congestion (black star), and numerous pyknotic nuclei (black arrow)

Histopathological examinations in (Fig. 6a and b) demonstrate that C-group showed normal central vein surrounded by normal hepatocytes which arranged in cords and separated by blood sinusoids; T-group showed excessive periportal and inter lobular fibrosis (black star), loss general architecture of hepatic lobules, mononuclear cell infiltration (black arrowhead), binucleated hepatocytes (black arrow), and pyknotic nuclei undergoing apoptosis (white arrow). Moreover, T-M group showed no fibrosis but a small number of pyknotic nuclei and a little periportal degeneration of hepatocytes with mononuclear cells infiltration; T-E group showed a small number pyknotic nuclei (white arrow) and mononuclear cells infiltration (black arrow) (Fig. 6c and d). T-EM group showed little periportal fibrosis (black arrow), pyknotic nuclei (white arrowhead), blood vessel congestion (black star), binucleated hepatocytes (white arrow), and mononuclear cell infiltration (black arrowhead) (Fig. 6e). M-TM-M group showed pyknotic nuclei (white arrow), blood vessel congestion (black star), and few centrilobular degeneration of hepatocytes (black arrow) and mononuclear cells infiltration (black arrowhead). Additionally, M-group showed only blood vessel congestion (black star) and numerous pyknotic nuclei (black arrow) (Fig. 6f and g).

## Discussion

Epidemiological studies have revealed that phenolics (including flavonoids)-rich diets decrease mortality caused by degenerative diseases resulting by the OS (Pandey and Rizvi 2009). Our results showed that M contains high content of total phenolics and total flavonoids where their analysis showed the presence of chlorogenic, caffeic, 2,5-dihydroxy benzoic, 3,5-dicaffeoylquinic, 4,5-dicaffeoylquinic, tannic and cinnamic acids, and catechin, phloridzin ,and quercetin. Additionally, M contains mangiferin, kaempferol, and gallotannins (Masibo and He 2009). Furthermore, M is a rich source of vitamins A and C. All these compounds have antioxidant activity (Lauricella et al. 2017), and this confirmed by our results which showed that M has high total antioxidant capacity. So, in this current study, the protective and therapeutic role of M and the drug E, separately, was evaluated against rat hepatotoxicity caused by T. Also, the therapeutic role of M and E together against hepatotoxicity was evaluated to determine the synergistic effect.

The current histopathological results revealed that T administration induced hepatotoxicity resulting in liver damage and this was confirmed by the disturbance of liver functions since the liver damage led to the leakage of its enzymes into the blood circulation. So, AST, ALT, GGT, and bilirubin levels in serum were elevated as compared to the C group, while STP, albumin, and LTP were reduced. Furthermore, our results showed that T administration caused elevations in MDA and GSSG levels beside GST and GSR activities as compared with the C group, but reduced GSH level and redox status (GSH/GSSG), and GPx and SOD activities. This indicates that T induced hepatotoxicity through the induction of OS. The increase in MDA content is an important index of lipid peroxidation, and this may be due to an enhanced generation of free radicals, which accelerated peroxidation of native membrane lipids especially polyunsaturated fatty acids (Shaban et al. 2003, 2017). Peroxidation of membrane lipids leads to loss of cell integrity, increasing in membrane permeability and alteration of Ca^+2^ homeostasis and change in the inner membrane potential causing cell damage (Shaban et al. 2003). The mechanism of hepatotoxicity by T may be due to the toxic effect of T and its reactive metabolites (T-S-oxide and T-S,S-dioxide radicals). These radicals accelerate the peroxidation of membrane lipids leading to the damage of the cells and their intracellular organelles (Friedman 1999; Saed et al. 2017; Shaban et al. 2017). Otherwise, GSH acts as a cofactor to some enzymes including GPx as well as it acts as nucleophilic scavenger of several active compounds by chemical and enzymatic mechanisms. So, the reduction in GSH level after T administration may be due to increase its demand for lipid hydroperoxide metabolism by GPx. Also, the diminishing GSH level may be due to its interaction with any free radicals, including T-S-oxide and T-S, S-dioxide. GSR plays an important role in cellular protection against OS by reducing GSSG accumulation of and thus maintaining the redox state. So, the increase in GSR activity after T administration possibly reflects an adaptation to oxidative circumstances (Shaban et al. 2003). GST is a versatile system involved in xenobiotics detoxification. It protects the cell against electrophilic radicals through catalyzing the conjugation of GSH with radicals via thioether linkages forming glutathione-S-conjugates leading to increase GST activity (Sanz et al. 2002). Furthermore, T, T-S-oxide and T-S,S-dioxide may increase GST gene expression through specific receptors, which are up-regulated by inflammatory and fibrotic agents including TNFα and TGF-β (Higgins and Hayes 2011). Conversely, the decrease in SOD and GPx activities may be related to their inhibition by T and its metabolites through direct interaction with the enzyme molecules or modification of the post-transcriptional and post-translational steps in enzyme synthesis (Hayakawa and Kuzuya 1990). Also, SOD inhibition may be associated with the oxidation of its cysteine residues (Shaban et al. 2014). Additionally, GSH depletion leads to the reduction of GPx activity (Chen et al. 2015). Otherwise, TNF-α is a major pro-inflammatory cytokine involved in early inflammatory events. It triggers a series of various inflammatory molecules, including other cytokines, chemotactic cytokines, and chemokines, such as interleukin 8. Otherwise, PDGF-BB is one of numerous growth factors that regulate cell growth and division. PDGF-BB plays a significant role in blood vessel formation and proliferation of mesenchymal cells such as fibroblasts. Our results showed that T administration caused elevations in 15 levels beside up-regulation of the TGF-ß1 gene expression level when compared with the C group. This indicates that T induced liver inflammation and fibrosis, and this may be linked to the increase in lipid peroxidation. The lipid peroxidation induces over expression of cytokines and increases the synthesis of collagen which activates hepatic stellate cells (HSCs) (Rejitha et al. 2012; Ulla et al. 2017). The activated HSCs and immune cells induce production and secretion of TGF-β1 and PDGF-BB where the overproduction of TGF–β1 is a major cause of tissue fibrosis (Habtemariam 1997; Park et al. 2016).

In contrast, treatment of rat hepatotoxicity with M [(before, during and after) or after T administration] or E or ME improved liver architecture where the microscopic examination showed that there were no fibrosis. but there were small numbers of pyknotic nuclei undergoing apoptosis. Also, we noticed that the histopathology and biochemical results are in harmony with each other. Since all these treatments improved the liver functions as the levels of AST, ALT, and GGT and bilirubin became lower than the T group, while TP and albumin in serum and hepatic TP became greater than the T group. Also, these treatments decreased OS, lipid peroxidation, liver inflammation, liver fibrosis, and liver injuries induced by T. The levels of MDA and GSSG and the activities of GST and GR were decreased, but GSH level and the activities of SOD and GPx were increased indicating that M and E have antioxidant activity. Furthermore, these treatments caused reduction in the levels of TNF-α, PDGF-BB, and TGF-β1 expression which became lower than the T group and this signifying that M and E have anti-inflammatory and anti-fibrotic activities. Otherwise, the present results revealed that treatment with M before, during, and after T administration (i.e., protective role) gave better results than treatment with M after the administration of T (i.e., therapeutic role). The mechanism of the protective and therapeutic effects of M may be related to the antioxidant activity of its contents, including phenolics and flavonoids and their gradients, carotenoids, vitamins, and some minerals. The antioxidant activities of phenolic and flavonoid compounds may be due to their ability to scavenge the ROS and their capability to up-regulate non-enzymatic and enzymatic antioxidants (López et al. 2007; Mehra et al. 2013; Abdel-Rahman et al. 2015; Shaban et al. 2016b; Ulla et al. 2017). Additionally, carotenoids, vitamins as A and C, and some minerals as Zn and Cu play important roles as antioxidants. Cu is a principal component of SOD and has a vital role in the synthesis of antioxidant protein called ceruloplasmin (Shazia et al. 2012).

The anti-inflammatory and anti-fibrotic effects of M are probably due to the presence of phenolics, flavonoids, and their gradients. Phenolics and flavonoids, including quercetin, catechin, chlorogenic acid, and mangiferin, attenuate TNF-α-induced expression of inflammatory genes and signaling pathways, suppress TGF-β-induced collagen production, and induce a apoptosis (Masibo and He 2009; Islam et al. 2014; Ooi et al. 2016; Shaban et al. 2016a; Santana-Gálvez et al. 2017). In addition, the reduction of OS led the reduction of TNF-α, PDGF-BB, and TGF-β1 expression. It has been reported that the inhibitors of TGF–β1 may be clinically useful as anti-fibrotic agents (Rejitha et al. 2012; Ulla et al. 2017).

On the other hand, our results showed the reduction in OS, lipid peroxidation, and liver damage in rats treated with E after T administration indicating that E has antioxidant power. These results reconcile with Labiós et al. (2008) who reported that E corrects the oxidative disturbances in hypertensive patients via the reduction of ROS and reactive nitrogen species and quenching the free radicals. Also, the anti-inflammatory and anti-fibrotic effects of E perhaps owed to its ability to control the increasing levels of Ang II, TGF-β, and collagen expressions. These results are in agreement with the results of the previous studies (Labiós et al. 2008; Masibo and He 2009). Unfortunately, treatment with M and E together after T administration gave the lower impact than the treatment with M or E in all markers of the OS, inflammation, fibrosis, and liver functions. The data showed that the CI values for the M and E co-administration on major tested parameters are greater than one. This points that there is anti-synergistic effect between M and E, and this may be related to the effect of the different gradients of M. These gradients may decrease the absorption of E, or may decrease the binding of E with the blood protein (albumin), or may decrease the binding of E with its receptor. In addition, we suggest that some M gradients may bind with E forming inactive product. All these probabilities may decrease the bioavailability of each E and M as antioxidant, anti-inflammatory, and anti-fibrotic (Rodríguez-Fragoso and Reyes-Esparza 2013).

Otherwise, the administration of healthy rats with M for 12 weeks showed non-significant changes in histopathological results and the markers of the OS, inflammation, fibrosis, and liver functions when compared with the C group. These changes may be due to the accumulation of some polyphenolics and Fe and Ca ions in liver tissues, where the phenolics and flavonoids are metabolized by peroxidases in the presence of Fe and Ca ions forming free radicals (Shazia et al. 2012; Shaban et al. 2013). These observations are agreeing with previous studies (Shaban et al. 2014, 2017).

## Conclusion

M and E revealed their therapeutic effect against hepatotoxicity induced by T by diminishing the oxidative stress, inflammation, and fibrosis. Also, M showed its protective role against hepatotoxicity where the treatment with M before, during, and after T administration (group M-TM-TM) gave a better effect than its treatment after T administration (group T-M). Otherwise, treatment of hepatotoxicity using M and E showed less effectiveness than the treatment with M or E, separately, and this indicates the presence of anti-synergistic effect between them. The administration of healthy rats with M for 12 weeks has no side effect.

## Data Availability

All data generated or analyzed during this study are included in this published article.
